# Two-component regulatory systems in *Pseudomonas aeruginosa*: an intricate network mediating fimbrial and efflux pump gene expression

**DOI:** 10.1111/j.1365-2958.2010.07527.x

**Published:** 2011-01-24

**Authors:** Melissa Sivaneson, Helga Mikkelsen, Isabelle Ventre, Christophe Bordi, Alain Filloux

**Affiliations:** 1 Imperial College London, Division of Cell and Molecular Biology, Centre for Molecular Microbiology and InfectionSouth Kensington Campus, Flowers Building, SW7 2AZ London, UK; 2 Laboratoire d'Ingénierie des Systèmes Macromoléculaires, CNRS – Aix Marseille Université31 Chemin Joseph Aiguier, 13402 Marseille, France

## Abstract

*Pseudomonas aeruginosa* is responsible for chronic and acute infections in humans. Chronic infections are associated with production of fimbriae and the formation of a biofilm. The two-component system Roc1 is named after its role in the regulation of c*up* genes, which encode components of a machinery allowing assembly of fimbriae. A non-characterized gene cluster, *roc2*, encodes components homologous to the Roc1 system. We show that cross-regulation occurs between the Roc1 and Roc2 signalling pathways. We demonstrate that the sensors RocS2 and RocS1 converge on the response regulator RocA1 to control *cupC* gene expression. This control is independent of the response regulator RocA2. Instead, we show that these sensors act via the RocA2 response regulator to repress the *mexAB-oprM* genes. These genes encode a multidrug efflux pump and are upregulated in the *rocA2* mutant, which is less susceptible to antibiotics. It has been reported that in cystic fibrosis lungs, in which *P. aeruginosa* adopts the biofilm lifestyle, most isolates have an inactive MexAB-OprM pump. The concomitant RocS2-dependent upregulation of *cupC* genes (biofilm formation) and downregulation of *mexAB-oprM* genes (antibiotic resistance) is in agreement with this observation. It suggests that the Roc systems may sense the environment in the cystic fibrosis lung.

## Introduction

Bacteria constantly probe the surrounding environment to adapt their colonization strategy be it in the environment or within a host. An important molecular device to achieve sampling of environmental signals is the so-called two-component regulatory system (TCS) (Stock *et al*., 2000). This system consists of a sensor, which is a histidine kinase capable of autophosphorylation on a conserved histidine residue, and a response regulator on which the phosphate is transferred. The phosphate is loaded onto a conserved aspartate residue in the conserved receiver domain of the response regulator, and the phosphorylation event results in activation of the output domain of the regulator. Frequently, the output domain is a DNA binding domain, which contributes directly to the control of gene expression. In some cases, the output domain carries an enzymatic activity (Galperin and Nikolskaya, 2007).

The distribution of TCSs in bacterial genomes is quasi-universal but the number of genes encoding TCSs can vary from a few to hundreds. Obviously, the more the bacterial strain copes with a complex environment, or is versatile, the more TCSs are needed to contribute to its adaptation. This is possible because histidine kinases have extremely variable detection or input domains, which are involved in the recognition of a specific stimulus (Galperin and Nikolskaya, 2007). Environmental signals can be related to oxygen availability, nutrient limitation, phosphate limitation or osmolarity, but sensors can also detect antimicrobial peptides (Otto, 2009), signalling molecules such as homoserine lactones (Freeman and Bassler, 1999) and many as yet unknown molecules. However, despite the variability of the input domain, the phosphorylation cascade resulting from the detection of the signal is a conserved process, and the structure and sequence of the catalytic machinery found in histidine kinases displays high similarity. Similarly, the receiver domain of response regulators is highly conserved.

*Pseudomonas aeruginosa* belongs to a category of versatile bacteria that encounter different environments, infect various hosts and have a broad catabolic potential. The PAO1 genome sequence analysis revealed the presence of about 130 genes encoding TCS components (Rodrigue *et al.*, 2000). In theory, a signalling cascade is very specific, with one sensor talking to its cognate response regulator. This specificity contributes to the avoidance of unwanted cross-talk between TCSs that may result in inappropriate adaption (Laub and Goulian, 2007; Skerker *et al*., 2008). The molecular basis of this specificity is becoming to be more extensively understood, and involves a limited number of key residues involved in the interaction between sensors and regulators (Skerker *et al*., 2008; Casino *et al*., 2009). However, one could imagine that complex environmental signals could be characterized by a series of sensors that feed multiple signals into one single response regulator, and thus provide an integrated response. Among the many *P. aeruginosa* TCSs, only a few have been characterized in great detail, and the signalling molecules have not always been identified. A large number of signalling pathways involving chemotaxis (Garvis *et al*., 2009) and TCSs (Gooderham and Hancock, 2009) have been described as having a key role during the infection process in *P. aeruginosa*. The GacAS/LadS/RetS system is an example of a regulatory pathway for which the signal is unknown (Kay *et al*., 2006; Ventre *et al*., 2006; Goodman *et al*., 2009). It constitutes an intricate network of several sensors, and is a major player in the control of *P. aeruginosa* virulence. In particular, the RetS and GacS sensor signalling pathways terminate on the GacA response regulator, which in turn modulates expression of small RNAs (Brencic *et al*., 2009). The input is thus variable but the output is conserved, with a final antagonistic control on genes involved in biofilm formation, such as the *pel* genes (Vasseur *et al*., 2005), and genes involved in virulence and cytotoxicity such as the type III secretion genes. Another *P. aeruginosa* signalling pathway, for which the signal is unknown, is the Roc1 system (Kulasekara *et al*., 2005). This time, one sensor, RocS1, feeds onto two response regulators, RocA1 and RocR. In this case the detection of a single signal may lead to a diversified response. The Roc system is also a major player in controlling the balance between biofilm formation and cytotoxicity (Kuchma *et al*., 2005). In particular, it positively controls the expression of *cup* genes involved in fimbrial assembly (Ruer *et al*., 2007), and negatively controls genes involved in the type III secretion system (T3SS) (Kuchma *et al*., 2005). Interestingly, there are additional sensors, which are coded by *rocS1* paralogous genes, namely *rocS2* and *rocS3* (Kulasekara *et al*., 2005).

In the present study we analysed the potential cross-regulation between these systems and how this impacts *P. aeruginosa* gene expression. We demonstrated that RocS1 and RocS2 could signal to both the RocA1 and RocA2 response regulators. However, the responses arising from the activation of either response regulator are totally different. Our analysis further highlights an intriguing adaptive combination, which suggests that biofilm formation and antibiotic resistance could be controlled antagonistically.

## Results

### RocS2 and RocS1 control *cupC* gene expression in a RocA1-dependent but RocA2-independent manner

We previously reported that transposon insertions within the *P. aeruginosa PA3946-PA3948* genetic locus, or *roc1* locus, resulted in elevated *cupC* gene expression (Kulasekara *et al*., 2005; Ruer *et al*., 2007). This upregulation resulted from overproduction of the sensor kinase RocS1 (PA3946) or the response regulator RocA1 (PA3948), a TCS (Fig. S1). In the same study, it was also shown that transposon insertions in the intergenic region of the *PA3044-PA3045* locus, or *roc2* locus, increased *cupC* gene expression levels. It is noticeable that the sensor kinases RocS1 (PA3946) and RocS2 (PA3044) are paralogues (45% identity), likewise with the pair of response regulators RocA1 (PA3948) and RocA2 (PA3045) (59% identity) (Fig. S1). The *roc1* locus also codes for RocR (PA3947), a response regulator with an EAL-containing C-terminal domain identified as a phosphodiesterase output domain (Fig. S1) (Rao *et al*., 2008). It has previously been shown that RocR antagonizes the activity of RocA1 (Kulasekara *et al*., 2005).

**Fig. 1:**
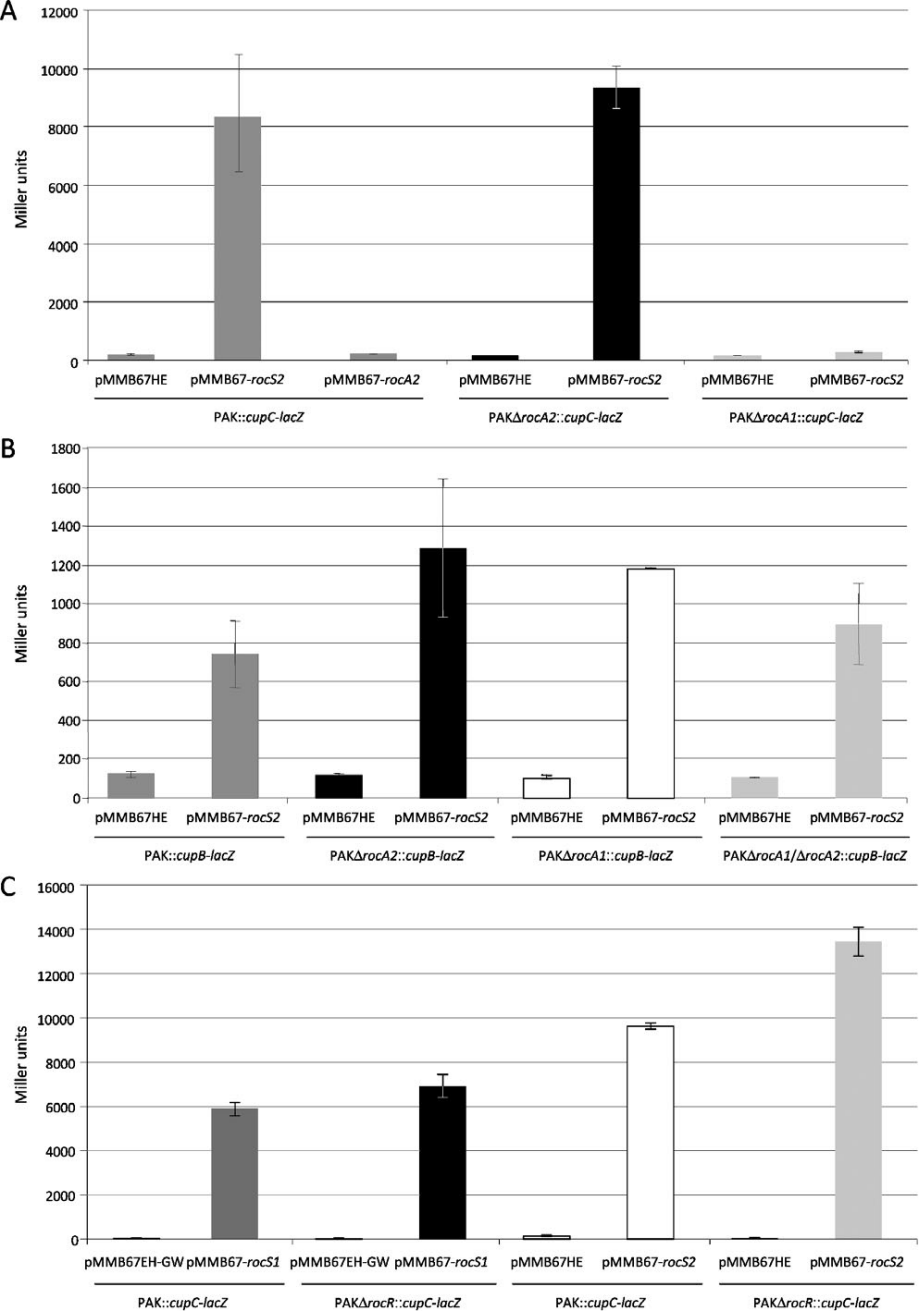
β-galactosidase activity (Miller Units) of PAK wild type or isogenic deletion mutants carrying chromosomal transcriptional fusions at the *attB* site (*cupB-lacZ* or *cupC-lacZ*) as well as a pMMB67HE vector, either empty or overexpressing *rocS2, rocA2* or *rocS1* as indicated. Data are the average of biological duplicates, and error bars indicate one standard deviation of the mean. A. *rocS2* overexpression induces *cupC* expression, but this induction is not seen by overexpressing *rocA2*. The difference between the strain containing the pMMB67HE vector or containing pMMB67-*rocS2* is significant with a *P*-value < 0.001. Furthermore, *rocS2* still induces *cupC* expression in the PAKΔ*rocA2* mutant (difference between the strain containing the pMMB67HE vector or containing pMMB67-*rocS2* is significant with a *P*-value < 0.001), but the induction is lost in the PAKΔ*rocA1* mutant indicating that RocA1, but not RocA2, is required for this signalling. B. *rocS2* overexpression also induces *cupB* expression. This induction is independent of both RocA2 and RocA1 as seen in the single and double deletion mutants. All differences between the strain containing the pMMB67HE vector or containing pMMB67-*rocS2* are significant with a *P*-value < 0.05. C. *cupC* expression can be induced by both *rocS1* and *rocS2*, but in both cases the promoter activity is higher in a PAKΔ*rocR* mutant showing that RocR is a repressor of *cupC* gene expression. The difference between the strains PAK::*cupC-lacZ* and PAKΔ*rocR*::*cupC-lacZ* containing pMMB67-*rocS2* is significant with a *P*-value < 0.02.

In this study, we further analysed the role of the Roc2 system. We cloned the *rocS2* and *rocA2* genes into the broad host range vector pMMB67HE42, yielding pMMB67-*rocS2* and pMMB67-*rocA2* respectively (Table 1). We then introduced either one of the recombinant plasmids into the *P. aeruginosa* PAK strain containing a *cupC-lacZ* transcriptional fusion at the chromosomal *attB* site (Table 1). Strikingly, overproduction of RocS2 in this strain resulted in a 40-fold increase in β-galactosidase activity, as compared with the strain harbouring the vector control, pMMB67HE (Fig. 1A). In contrast, overproduction of RocA2 from pMMB67-*rocA2* had no effect on *cupC-lacZ* transcription (Fig. 1A). In order to verify that RocA2 has no role in *cupC* gene expression, we engineered a *rocA2* deletion mutant in the PAK strain, PAKΔ*rocA2* (Table 1), and introduced the *cupC-lacZ* fusion into the chromosome. Upon RocS2 overproduction, induction of *cupC* gene expression was still observed in the *rocA2* mutant (Fig. 1A). This confirmed that RocA2 is not involved in the induction of *cupC* genes by RocS2. Because RocA2 and RocA1 are paralogues, we then investigated whether RocS2 could act through RocA1 to activate *cupC* expression. As for *rocA2*, we engineered a *rocA1* mutant in the PAK strain carrying the *cupC-lacZ* fusion. Upon introduction of pMMB67-*rocS2* into the *rocA1* mutant, no activation of the fusion could be observed and β-galactosidase levels were similar to those observed in a strain containing the vector control (Fig. 1A). Thus, we concluded that *cupC* gene expression occurs through RocA1 not only upon activation by RocS1 (Kulasekara *et al*., 2005) but also via RocS2 signalling.

**Table 1:** Bacterial strains and plasmids used in this work

Strains/Plasmid	Relevant characteristicsa	Source/Reference
Strain		
*Escherichia coli*		
TG1	*supE*Δ(*lac-proAB*) *thi hsdR*Δ*5* (F' *traD36 rpoA*^+^*B*^+^*lacI*^q^*Z*ΔM15)	Lab collection
SM10 (λpir)	*thi*-1 *thr*-1 *leu*B6 *sup*E44 *ton*A21 *lac*Y1 *rec*A^-^::RP4-2*-Tc*::*Mu* Km^r^	Lab collection
TOP10F'	F'[*lacI*^q^ Tn10(*tetR*)]*mcrA*Δ(*mrr-hsdRMS-mcrBC*) Φ80*lacZ*ΔM15 Δ*lacX*74 *deoR nupG recA1 araD139*Δ (*ara-leu*)7697 *galU galK rpsL* (Str^r^) *endA1*λ	Invitrogen
CC118 (λpir)	Host strain for pKNG101 replication; Δ(*ara-leu*) *araD*Δ*lacX74 galE galK phoA20 thi-1 rpsE rpoB argE*(Am) *recA1* Rf^r^ (λpir)	Lab collection
DHMI	*cya-854 recA1 gyrA96* (NaI) *thi1 hsdR17 spoT1 rfbD1 glnV44*(AS)	Karimova *et al*. (1998)
*Pseudomonas aeruginosa*		
PAK	Wild type	D. Bradley
PAKΔ*rocA1*	In frame deletion of *rocA1* (*PA3948*) in PAK	Kulasekara *et al*. (2005)
PAKΔ*rocR*	In frame deletion of *rocR* (*PA3947*) in PAK	Kulasekara *et al*. (2005)
PAKΔ*rocA2*	In frame deletion of *rocA2* (*PA3045*) in PAK	This study
PAKΔ*rocA1-rocA2*	In frame deletion of *rocA1* (*PA3948*) and *rocA2* (*PA3045*) in PAK	This study
PAK::*cupC-lacZ*	PAK with a *cupC-lacZ* fusion integrated on the chromosome at the *attB* site	This study
PAKΔ*rocA1*::*cupC-lacZ*	*rocA1* deletion mutant in PAK::*cupC-lacZ*	This study
PAKΔ*rocA2::cupC-lacZ*	*rocA2* deletion mutant in PAK::*cupC-lacZ*	This study
PAKΔ*rocR::cupC-lacZ*	*rocR* deletion mutant in PAK::*cupC-lacZ*	This study
PAK::*cupB-lacZ*	PAK with a *cupB-lacZ* fusion integrated on the chromosome at the *ctx att* site	This study
PAKΔ*rocA1*::*cupB-lacZ*	*rocA1* deletion mutant in PAK::*cupB-lacZ*	This study
PAKΔ*rocA2*::*cupB-lacZ*	*rocA2* deletion mutant in PAK::*cupB-lacZ*	This study
PAKΔ*rocA1*-*rocA2*::*cupB-lacZ*	*rocA1-rocA2* double deletion mutant in PAK::*cupB-lacZ*	This study
Plasmid		
pRK2013	ColE1 *mob*+*tra*_RK2_Δ*rep*_RK2_*repE*^-^ Km^r^	Figurski and Helinski (1979)
pCR2.1	TA cloning vector for PCR products, *lacZ*α ColE1 f1 *ori* Ap^r^ Km^r^	Invitrogen
pMMB67HE	Broad host range vector, *tac* promoter Ap^r^	Lab collection
pMMB67EH-GW	Broad host range vector, *tac* promoter Gm^r^	Lab collection
pET-Dest42	Destination vector for gateway technology, T7 promoter, Ap^r^	Invitrogen
pMMB67HE42	pMMB67HE containing the gateway cassette from pET-Dest42 Ap^r^	E. Termine
pMMB67-*rocS2*	pMMB67HE harbouring the *rocS2* gene from the Gateway library	This study
pMMB67-*rocA2*	pMMB67HE harbouring the *rocA2* gene from the Gateway library	This study
pMMB67-*rocS1*	pMMB67EH-GW harbouring the *rocS1* gene from the Gateway library	Kulasekara *et al*. (2005)
pKNG101	Suicide vector for *P. aeruginosa*. *sacB*, Sm^r^	Kaniga *et al*. (1991)
pKNGΔ*rocA2*	pKNG101 containing 560 bp upstream and 505 bp downstream from the *rocA2* gene DNA fragment	This study
miniCTX-*lacZ*	Vector for unmarked integration into *P. aeruginosa att* site. oriT, FRT, int, Tc^r^	Hoang *et al*. (2000)
miniCTX-*cupC-lacZ*	*cupC1* promoter cloned into miniCTX-*lacZ* (*Bam*HI/*Xho*I)	This study
miniCTX-*cupB-lacZ*	*cupB1* promoter cloned into miniCTX-*lacZ* (*Bam*HI/*Xho*I)	This study
pKT25	Cloning and expression vector, encodes the T25 fragment (amino acids 1–224 of CyaA), Km^r^	Karimova *et al*. (1998)
pUT18c	Cloning and expression vector, encodes the T18 fragment (amino acids 225–399 of CyaA), Ap^r^	Karimova *et al*. (1998)
pKT25-*torR*	DNA fragment encoding the D2 domain of TorR cloned into pKT25	Kulasekara *et al*. (2005)
pUT18c-*torS*	DNA fragment encoding the Hpt domain of TorS cloned into pUT18c	Kulasekara *et al*. (2005)
pKT25-*rocS1*	DNA fragment encoding the Hpt domain of RocS1 cloned into pKT25	Kulasekara *et al*. (2005)
pUT18c-*rocA1*	DNA fragment encoding the D2 domain of RocA1 cloned into pUT18c	Kulasekara *et al*. (2005)
pUT18c-*rocR*	DNA fragment encoding the D2 domain of RocR cloned into pUT18c	Kulasekara *et al*. (2005)
pKT25-*rocS2*	DNA fragment encoding the Hpt domain of RocS2 cloned into pKT25	This study
pUT18c-*rocA2*	DNA fragment encoding the D2 domain of RocA2 cloned into pUT18c	This study
pUT18c-*trpO*	DNA fragment encoding the D2 domain of PA0034 cloned into pUT18c	This study
pKT25-*gacS*	DNA fragment encoding the H1-D1-Hpt domain of GacS cloned into pKT25	This study
pUT18c-*gacA*	DNA fragment encoding the D2 domain of GacA cloned into pUT18c	This study

a Ap^r^, ampicillin resistance; Sm^r^, Streptomycin resistance; Km^r^, Kanamycin resistance, Tc^r^, Tetracycline, Gm^r^, Gentamicin resistance.

### RocS2 and RocS1 control *cupB* gene expression in a RocA1- and RocA2-independent manner

It was previously shown that RocS1 not only activates *cupC* gene transcription but also influences *cupB* gene expression. We engineered a series of *P. aeruginosa* PAK strains containing a *cupB-lacZ* transcriptional fusion at the chromosomal *attB* site, PAKΔ*rocA1*, PAKΔ*rocA2* or a double mutant PAKΔ*rocA1−rocA2* (Table 1). As previously shown with RocS1 (Kulasekara *et al*., 2005), overproduction of RocS2 resulted in increased levels of *cupB* gene expression by about sixfold (Fig. 1B). However, and in contrast to what was observed with the *cupC* genes, upregulation of *cupB* gene expression persisted in the *rocA1*, *rocA2* and even *rocA1-rocA2* double mutant (Fig. 1B). Similar results were obtained when the plasmid carrying the *rocS1* gene (pMMB67-*rocS1*) was introduced in these different strains (Fig. S2). This was unexpected and suggested that induction of *cupB* gene expression *via* the RocS1 and RocS2 signalling pathways is independent of both RocA1 and RocA2. It is thus obvious that additional components are likely to be part of the Roc regulatory pathway, and the regulatory component responsible for *cupB* gene control is yet to be identified.

### RocR is a negative regulator of the Roc1 and Roc2 systems

The RocR response regulator has a phosphodiesterase activity (Rao *et al*., 2008) and was shown previously to have an antagonist effect as compared with RocA1, as RocR represses *cupC* gene expression (Kulasekara *et al*., 2005). We tested whether the negative effect could be exerted whether the input came from either RocS1 or RocS2. We engineered a *rocR* mutant strain (PAKΔ*rocR*) in which the transcriptional *cupC-lacZ* fusion was inserted at the *attB* site on the chromosome (Table 1). We then introduced a plasmid carrying either *rocS1* or *rocS2*. In both cases we observe that the induction of *cupC* gene expression was significantly higher in the *rocR* mutant background as compared with the parental strain (Fig. 1C). We thus concluded that RocR is a negative regulator not only of the Roc1 system but also of the Roc2 system. This observation further expands the cross-regulation between the Roc1 and Roc2 systems.

### RocS1/A1 and RocS2/A2 form a complex and interactive network

**Fig. 2:**
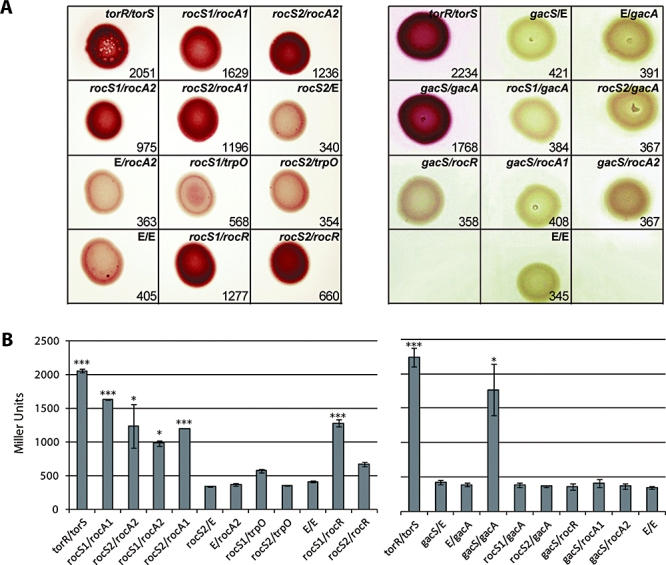
Protein–protein interactions between components of the Roc1 and Roc2 systems as determined by bacterial two-hybrid analysis. Various combinations of recombinant pKT25 and pUT18c plasmids harbouring protein domains of interest (Hpt domains of sensors and D2 domains of response regulators) were co-transformed into *E. coli* DHM1, and transformants were spotted onto MacConkey agar (A). Dark red colonies indicate a positive interaction. The *E. coli* two component sensor TorS, together with its cognate response regulator TorR, was used as a positive control. The specificity of interaction was assessed using the unrelated *P. aeruginosa* TCS GacS/GacA, as well as the unrelated *P. aeruginosa* response regulator TrpO. The strength of interaction was investigated by measuring the β-galactosidase activity of cells in the respective colonies, and the average activity in Miller Units is indicated next to each colony. (B) Graphic representation of the β-galactosidase activity in panel A. All experiments were carried out in at least duplicates, and error bars represent one standard deviation of the mean. Plasmid combinations of pKT25/pUT18c, respectively, are indicated in panels A and B. It should be noted that E means empty vector. For specific constructs, see Table 1. In panel B, stars indicate a significant difference between a given combination and the control (E/E). Three stars or one star corresponds to *P*-value < 0.001 or < 0.05 respectively.

We previously reported that activation of RocA1 by RocS1 resulted from a direct interaction between these two components (Kulasekara *et al*., 2005). Because we identified cross-regulation between Roc1 and Roc2 in the control of *cupC* gene expression, we performed a systematic analysis to probe the interaction between Roc1 and Roc2 components using the *Escherichia coli* bacterial two-hybrid method, as previously described (Karimova *et al*., 1998; Kulasekara *et al*., 2005). Because RocS1 and RocS2 are unorthodox sensors, we cloned the DNA fragments corresponding to the Hpt domains (Fig. S1) into pKT25 (Karimova *et al*., 1998). We also cloned the fragments corresponding to the D2 receiver domain of RocA1 and RocA2 response regulators into pUT18c. We then tested tandem interactions between Hpt and D2 domains, and found that RocS1 and RocS2 could interact with both response regulators (Fig. 2). In order to validate this observation, which reveals an extensive cross-regulation between the Roc1 and Roc2 systems, we also used a control showing that neither RocS1 nor RocS2 could interact with the D2 domain of the unrelated response regulator TrpO/PA0034 (Ventre *et al*., 2004) (Fig. 2). We also tested a *P. aeruginosa* sensor kinase other than RocS1/RocS2, namely GacS. We showed that GacS strongly interacts with the cognate response regulator GacA, but no interaction is seen with either RocA1 or RocA2 (Fig. 2). We concluded that both RocS2 and RocS1 directly activate RocA1, which results in induction of *cupC* gene expression. Moreover, we also observed that both RocS1 and RocS2 interact with RocA2, which may result in the induction of as yet unknown target genes.

Finally, we previously observed that RocR acts as a negative regulator of the Roc1 system (Kulasekara *et al*., 2005) and we showed in this study that it also negatively impacts the Roc2 response. We cloned the fragment corresponding to the D2 receiver domain of RocR into pUT18c and tested the interaction with Hpt domains of RocS1 or RocS2. We confirmed the interaction between RocR and RocS1 and found that RocR is also able to directly interact with RocS2 (Fig. 2). This observation further explains the mechanism by which RocR negatively impacts RocS2 signalling.

### RocA2 controls expression of the *mexAB-oprM* genes

Because RocA2 is not involved in *cupC* gene expression, but interacts with RocS2 and RocS1, we further searched for specific RocA2 target genes. We compared the transcriptomic profile of a PAK or PAKΔ*rocA2* strain, both overproducing RocS2 from pMMB67-*rocS2*. This was based on the assumption that transcripts that were altered in PAK but not in PAKΔ*rocA2* are putative RocA2 target genes. The strains were grown at 37°C in minimal medium, RNA was extracted and cDNA synthesized and labelled using Cy5-dCTP. The cDNA was then hybridized to *P. aeruginosa* microarrays (see *Experimental procedures*). Gene expression levels in PAK overproducing RocS2 were directly compared with expression levels observed in PAKΔ*rocA2* overproducing RocS2. The genes for which expression varies by more than 1.5-fold are described in the *Supporting information* (Table S1). Using this cut-off, 42 genes were upregulated and 44 genes downregulated in the PAKΔ*rocA2* strain as compared with PAK. A number of genes involved in iron acquisition, such as *fepB*, *pchG* or *pvdH* are downregulated in PAKΔ*rocA2*, whereas at least five genes involved in T3SS are upregulated in PAKΔ*rocA2*, namely *pcr1*, *orf1*, *exoT*, *popB* and *pcrV* (Table S1). In all these cases, the fold change observed was between 1.5 and 1.9. Only eight genes were identified to be upregulated more than threefold, in PAKΔ*rocA2* (Table S1). Among these eight genes, four are located at a single locus. The *mexA, mexB* and *oprM* genes (around eightfold up in PAKΔ*rocA2*) encode components of an efflux pump whereas *mexR* encodes a regulator of *mexAB-oprM* gene expression (23-fold up in PAKΔ*rocA2*).

**Fig. 3:**
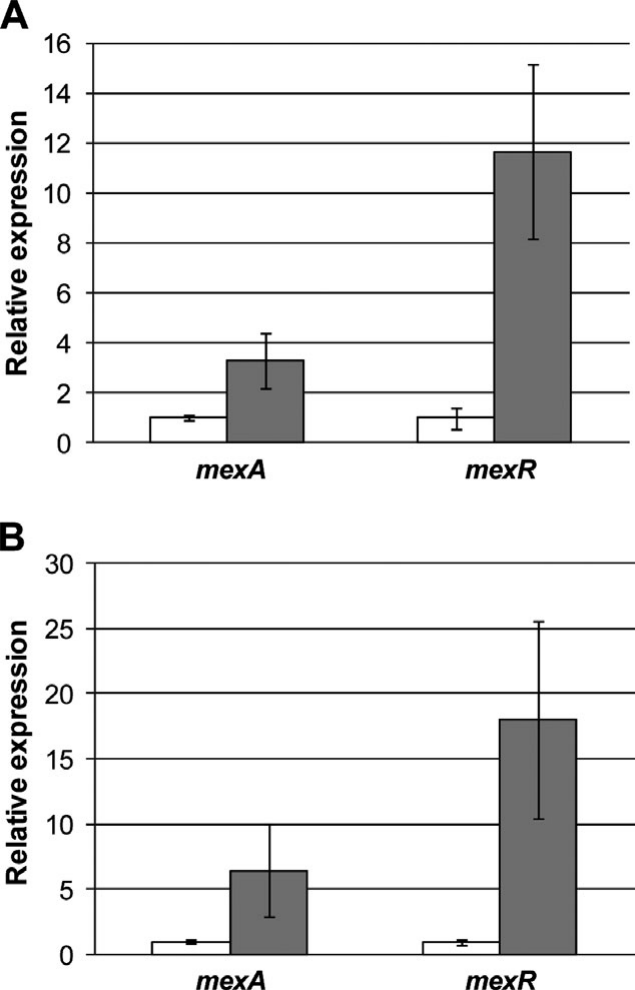
Quantitative RT-PCR analysis of *mexA* and *mexR* gene expression. The analysis was done upon overexpression of the sensor-encoding genes *rocS2* (A) and *rocS1* (B) in a PAK wild type (white bars) and PAKΔ*rocA2* deletion mutant (grey bars). Expression levels were normalized to the 16SrRNA gene and wild-type levels have been set to 1. The data represent the average of biological triplicates and error bars indicate one standard deviation of the mean. In (A) and (B) the relative expression of *mexA* is indicated on the left and *mexR* on the right, as indicated below the figure. In all cases the difference is significant with a *P*-value < 0.05.

In order to validate the microarray data, we performed quantitative RT-PCR (see *Experimental procedures*) on the *mexA* and *mexR* genes. Upon overexpression of *rocS2* we observed a threefold increase in *mexA* expression, and an almost 12-fold increase for *mexR* in PAKΔ*rocA2* as compared with PAK (Fig. 3A). These results confirmed that the *mexAB-oprM* and *mexR* genes are part of the RocA2 regulon, and that their expression is negatively controlled by RocA2. Interestingly, when we performed the experiment using *rocS1* overexpressing strains, we observed similar results. There is a sixfold and 17-fold upregulation in the PAKΔ*rocA2* strain for *mexA* and *mexR* respectively (Fig. 3B). We thus concluded that in addition to RocS2, RocS1 could also act on RocA2 to repress *mex* gene expression, which confirms the positive interaction observed between RocS1 and RocA2 using the two-hybrid approach.

### Roc2 is a signalling pathway involved in antibiotic resistance

**Fig. 4:**
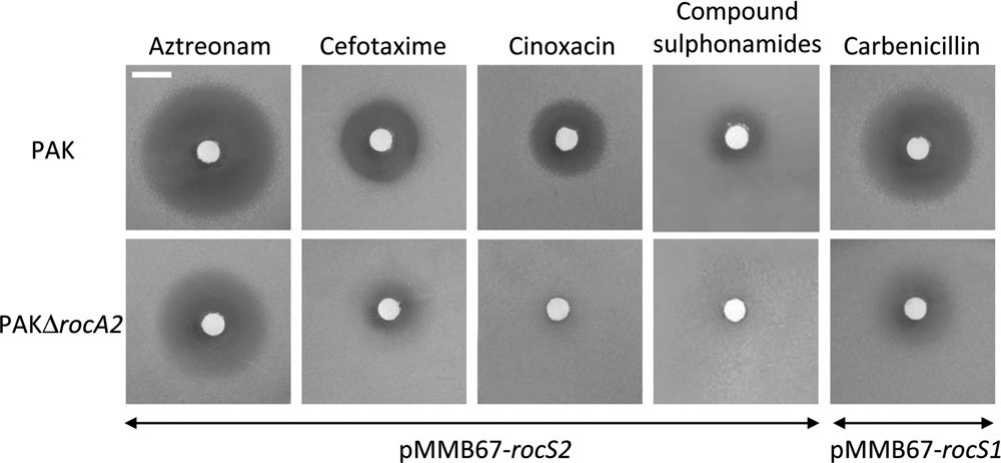
Antibiotic Disc Diffusion Assay. Discs containing Aztreonam (30 µg), Cefotaxime (300 µg) Cinoxacin (100 µg) or compound sulphonamides (300 µg) were applied to M63 media plates overlaid with PAK wild-type or PAKΔ*rocA2* harbouring pMMB67-*rocS2*. Discs containing Carbenicillin (100 µg) were applied to M63 media plates inoculated with PAK wild-type harbouring pMMB67-*rocS1* or PAKΔ*rocA2* harbouring pMMB67-*rocS1*. The bar in the top left panel is a 1 cm scale.

MexAB-OprM is a multidrug efflux system known to contribute to the natural resistance of *P. aeruginosa* to several antibiotics. As we showed that RocA2 could repress *mexAB-oprM* expression, we analysed whether this regulatory mechanism could impact the level of antibiotic resistance in *P. aeruginosa* strains. We used a series of antibiotic discs to assess the susceptibility of various *P. aeruginosa* strains (see *Experimental procedures*). We thus compared the susceptibility of PAK and PAKΔ*rocA2*, both overexpressing *rocS2*, for compound sulphonamides, cinoxacin and cefotaxime. Interestingly, the level of susceptibility to these antibiotics was decreased in the *rocA2* mutant as compared with the PAK strain, as seen by the diameter of bacterial growth inhibition around each disc (Fig. 4 and Table 2). We also determined the minimal inhibitory concentration (MIC) values for some of these antibiotics (see *Experimental procedures*) and showed that for the *rocA2* mutant, the MIC was twofold higher for cinoxacin and eightfold higher for sulfamethoxazole, which is a sulphonamide antibiotic, as compared with the PAK parental strain (Table 3). Finally, we used a specific substrate of the MexAB-OprM pump, namely the β-lactam aztreonam. Again, the *rocA2* mutant showed a significant increase in resistance to this antibiotic (Fig. 4 and Table 2). We also compared the antibiotic susceptibility of PAK or PAKΔ*rocA2*, both overexpressing *rocS1* from a plasmid that does not carry a carbenicillin resistance gene but has a gentamicin resistance cassette (pMMB67-*rocS1*). In this context we could show that the *rocA2* mutant has a decreased susceptibility towards the β-lactam carbenicillin, when compared with the parental PAK strain (Fig. 4 and Table 2). These observations confirm that the activity of RocA2 prevents MexAB-OprM efflux pump production, and therefore increases susceptibility to antibiotics, including β-lactams such as aztreonam and carbenicillin, which are specific substrates of the MexAB-OprM efflux pump. Consequently, in the absence of RocA2 the *P. aeruginosa* strains become hyper-resistant to antibiotics.

**Table 2:** Antibiotic susceptibility assays

	Zone of inhibition (mm)^a^				
	
Strain	Aztreonam (30 µg)	Cefotaxime (300 µg)	Cinoxacin (100 µg)	Sulphonamides (300 µg)	Carbenicillin (100 µg)
PAK + pMMB67-*rocS2*	15.5 ± 0.7	8 ± 0.2	8.5 ± 0.8	4 ± 0.8	ND
PAKΔ*rocA2* + pMMB67-*rocS2*	7.5 ± 0.4	3 ± 0.2	R^b^	R	ND
PAK + pMMB67-*rocS1*	15 ± 0.6	8 ± 0.1	7 ± 0.5	4 ± 0.7	13.5 ± 0.6
PAKΔ*rocA2* + pMMB67-*rocS1*	7.5 ± 0.3	2.5 ± 0.1	R	R	5.5 ± 0.7

a Zones of growth inhibition around discs were determined by the disc diffusion assay on M63 minimal media agar plates. The mean and standard deviation of a representative experiment with biological triplicates are given and the experiment has been repeated twice.

b R, resistant, no growth inhibition observed.

All differences observed between PAK/pMMB67-*rocS2* and PAKΔ*rocA2*/pMMB67-*rocS2* or PAK/pMMB67-*rocS1* and PAKΔ*rocA2*/pMMB67-*rocS1* are significant with a *P*-value < 0.01.

ND, Not determined.

**Table 3:** MIC values

	MIC (µg ml^−1^)^a^	
	
Strain	Cinoxacin	Sulfamethoxazole
PAK + pMMB67-*rocS2*	62.5	31.3
PAKΔ*rocA2* + pMMB67-*rocS2*	125	250

a MICs were determined for cinoxacin and sulfamethoxazole by a twofold broth dilution method and the lowest concentration where no visible growth was observed is indicated. The assay was done in triplicates.

## Discussion

*Pseudomonas aeruginosa* uses more than 60 different two-component signal transduction systems to adapt to various and changing environments (Gooderham and Hancock, 2009). A significant number of these systems are important for adaptation during the infection process. Among these the GacS/RetS/LadS/HptB pathway has been shown to be important in the switch between acute and chronic infections (Goodman *et al*., 2004; O'Toole, 2004; Ventre *et al*., 2006; Bordi *et al*., 2010). In the chronic infection process, it has been proposed that the development of a biofilm is the most appropriate bacterial lifestyle (Costerton, 2001). Biofilm formation requires several steps, including initial attachment to a surface, cell–cell interaction within microcolonies, formation of mushroom-like structures and finally bacterial dispersal, allowing the process to start all over again (O'Toole *et al*., 2000; Stoodley *et al*., 2002). Initial attachment onto a surface has been shown to involve Cup fimbriae (Vallet *et al*., 2001). However, the production and assembly of these cell surface appendages is tightly controlled and *cup* genes are typically not expressed in standard laboratory conditions (Kulasekara *et al*., 2005). It has further been shown that the Roc1 system is an important regulatory pathway in the production and assembly of CupB and CupC fimbriae (Ruer *et al*., 2007; 2008;). This system involves an unorthodox sensor, RocS1, and two response regulators. The output domain of RocA1 is a helix–turn–helix motif that should provide direct DNA binding properties, whereas the output domain of RocR contains an EAL domain, which is a signature of phosphodiesterase activity (Rao *et al*., 2008). It was shown that RocA1 positively controls *cup* gene expression, whereas RocR acts indirectly by modulating the intracellular pool of c-di-GMP. In *P. aeruginosa*, Roc1 paralogues could be identified, namely Roc2 and Roc3. Interestingly, all three histidine kinases, RocS1, RocS2 and RocS3, were identified as BvgS homologues, and BvgS is a global virulence regulator in *Bordetella pertussis* (Beier and Gross, 2008).

It has previously been reported that the Roc1 system controls the expression of *cupC* genes. This came from the identification of transposon insertions in the *roc1* region, which resulted in overexpression of *rocS1* or *rocA1* genes (Kulasekara *et al*., 2005). To a lesser extent, it was also shown that transposon insertions within the *roc2* cluster increased expression of *cupC* genes. Here we showed that it is indeed overexpression of the *rocS2* gene that results in *cupC* expression. However, it is intriguing that RocS2-dependent activation of *cupC* is totally independent of the RocA2 response regulator, but exclusively dependent on the RocA1 response regulator. This observation thus suggests the existence of cross-regulation between the Roc1 and Roc2 signalling pathways. Cross-regulation between TCSs is not frequent, at least *in vivo*, and bacteria have developed means to insulate pathways from unwanted cross-talk (Laub and Goulian, 2007). Importantly, and in contrast to *rocS1* and *rocS2*, the overexpression of *rocS3* did not result in *cupC* gene overexpression, suggesting a degree of specificity in the RocS1 or RocS2 interaction with RocA1.

It was intriguing to observe that RocA2 is not required for RocS2-dependent activation of *cupC* genes, because we showed using two-hybrid experiments that RocS2 is able to interact with RocA2, in addition to interaction with RocA1. The identification of the RocA2 regulon was thus completed using microarray analysis and we compared gene expression in a wild-type versus *rocA2* mutant upon overexpression of *rocS2*. Among the targets identified, it is noticeable that five T3SS genes were upregulated in the *rocA2* mutant. It is a possibility that T3SS genes are common targets both for RocA1 and RocA2, as previous observations by Kuchma and collaborators indicated that lack of both RocA1S1 (named SadAS in their study) resulted in slight derepression of the T3SS genes (Kuchma *et al*., 2005). Whereas the variation in T3SS gene expression was about 1.5-fold, we identified a series of target transcripts that varied much more, ranging from 3- to 12-fold. These genes, *mexAB-oprM* and *mexR* are involved in the regulation and assembly of an efflux pump that contributes to antibiotic resistance in *P. aeruginosa*. MexAB-OprM is one of the many pumps found in *P. aeruginosa* (Srikumar *et al*., 1999). It is likely the pump with the widest substrate specificity, because it is able to provide resistance towards β-lactams, fluoroquinolones and other families of antibiotics (Li *et al*., 1995; Masuda *et al*., 2000). The regulation of *mexAB-oprM* genes has already been extensively documented and underlines the existence of a number of pathways. MexR is the repressor that directly binds the *mexA* promoter region and prevents transcription of the *mexAB-oprM* operon (Evans *et al*., 2001). Interestingly, the *armR* gene (*PA3719*), encodes a 53 amino acids-long polypeptide that prevents proper binding of MexR within the *mexA* promoter region (Wilke *et al*., 2008). Another repressor, NalD, was also described, which acts by binding within another region of the *mexA* promoter (Morita *et al*., 2006). The reason why we observed an upregulation of the gene encoding the MexR repressor, and yet observed an upregulation of the *mexAB-oprM* operon is unclear. However, it should be noted that most *nalB* and *nalD* multidrug resistant mutants display an increased expression of both *mexR* and *mexAB-oprM* (Poole *et al*., 1996; Llanes *et al*., 2004). One possibility could be that the anti-repressor ArmR is also upregulated but we did not detect such variation in our microarray analysis. Alternatively, a gene encoding a yet unknown anti-MexR protein may be upregulated. MexR has also been reported to be sensitive to oxidative stress and oxidized MexR dissociates from the *mexA* promoter (Chen *et al*., 2008). It is a possibility that genes encoding components that contribute to oxidative stress conditions might also be upregulated.

One obvious strategy to analyse whether the observed upregulation of *mexAB-oprM* results in a relevant phenotype was to test the antibiotic susceptibility of the *P. aeruginosa rocA2* mutant overexpressing *rocS2* or *rocS1* as compared with the parental strain PAK. We have tested a series of antibiotics including sulphonamides and cinoxacin, but also β-lactams such as aztreonam and carbenicillin, which are specific substrates of the MexAB-OprM pump. In all cases the *rocA2* mutant showed a striking decrease in sensitivity as compared with the parental strain, which confirms that RocA2 acts as a repressor of *mexAB-oprM* gene expression. Regulation of antibiotic resistance by TCSs is not frequently observed in *P. aeruginosa*. However, it has been reported for CzcR/CzcS and CopR/CopS, which control metal and imipenem resistance (Hassan *et al*., 1999; Perron *et al*., 2004; Teitzel *et al*., 2006; Caille *et al*., 2007), PprB/PprA and GacA/GacS, which control aminoglycoside resistance (Brinkman *et al*., 2001; Wang *et al*., 2003), PmrA/PmrB involved in polymyxin B and antimicrobial peptide resistance (McPhee *et al*., 2003) or ParR/ParS involved in polymyxin B and colistin resistance (Fernández *et al*., 2010).

**Fig. 5:**
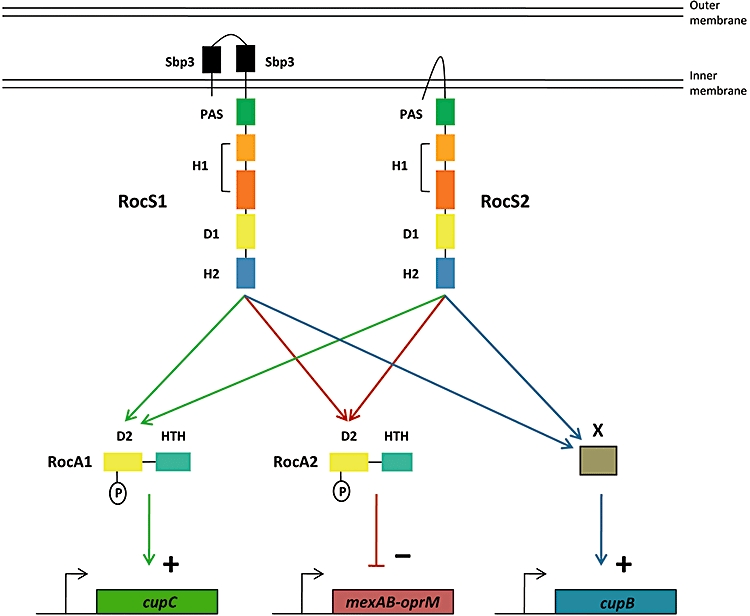
Schematic model showing the cross-regulation between Roc1 and Roc2 signalling pathways. The Roc1 and Roc2 components are represented as in Fig. S1. The RocS1 and RocS2 sensors are shown as integral inner membrane proteins. The positive regulation on the *cupC* gene expression is shown with green arrows. The negative regulation on the *mexAB-oprM* genes is shown with brown arrows. The positive regulation on *cupB* genes is shown with blue arrows and involves a yet unknown regulator indicated as X.

A striking feature here is the observation that the RocS2 and RocS1 sensors on one hand induce *cupC* gene expression and hence biofilm formation via RocA1, while on the other hand they repress antibiotic resistance mechanisms via RocA2. Although the opposition between cytotoxicity and biofilm formation observed through the RetS/LadS/GacS/HptB pathway appears plausible in the context of a chronic infection, the antagonistic regulation of biofilm formation and antibiotic resistance is less obvious. However, previous reports by De Kievit and collaborators have already suggested that efflux pumps may not play a role in the antibiotic resistance observed in *P. aeruginosa* biofilms (De Kievit *et al*., 2001). Using transcriptional fusions to *gfp* they demonstrated that genes encoding multidrug resistance pumps such as *mexAB-oprM*, were downregulated in the developing biofilm. Even more striking, a recent study by Vettoretti and collaborators suggested that a large proportion of the *P. aeruginosa* strains isolated from cystic fibrosis (CF) patients (28%) are hyper-susceptible to the β-lactam antibiotic ticarcillin (Vettoretti *et al*., 2009). In this study it was shown that the lack of resistance is essentially associated with a non-functional MexAB-OprM pump. In other words, in CF patients *P. aeruginosa* strains grow predominantly as biofilms in the respiratory tract (Bjarnsholt *et al*., 2009) and at the same time large numbers of these strains appear to become more susceptible to antibiotics. It is largely unclear what the benefit could be for this subpopulation, but likely the lack of this particular pump may provide a not yet understood advantage fitness in the context of CF lungs. Furthermore, other pumps may still provide reasonable levels of antibiotic resistance to cope with the selection pressure imposed by the antimicrobial treatments given to CF patients. Finally, other mechanisms have been shown to contribute biofilm resistance to antibiotics, such as the NdvB-dependent production of cyclic glucans (Mah *et al*., 2003; Sadovskaya *et al*., 2010). Nevertheless, our study fully supports the idea that an antagonistic balance exists between biofilm formation and antibiotic resistance in *P. aeruginosa*, and we show evidence that the TCSs Roc2 and Roc1 are likely to be master regulators in this process (Fig. 5). It also suggests that the Roc systems might be key regulatory systems sensing the particular environment of the CF lungs. In this respect, further studies aimed at the characterization of the stimuli detected by the Roc systems could be of great importance in the development of novel antimicrobials more effective in the treatment of CF patients.

## Experimental procedures

### Bacterial strains, plasmids and growth conditions

The bacterial strains and plasmids used are described in Table 1. Plasmids were transferred into *P*. *aeruginosa* using the conjugative properties of the helper plasmid pRK2013 (Figurski and Helinski, 1979) in triparental matings. *P. aeruginosa* transconjugants were isolated on Pseudomonas Isolation Agar (Difco) supplemented with the appropriate antibiotics. The following antibiotic concentrations were used for *E. coli*: ampicillin 50 µg ml^−1^, kanamycin 50 µg ml^−1^, streptomycin 50 µg ml^−1^, tetracycline 25 µg ml^−1^, gentamicin 25 µg ml^−1^ and for *P. aeruginosa*: carbenicillin 300 µg ml^−1^, streptomycin 2000 µg ml^−1^, gentamicin 120 µg ml^−1^, tetracycline 200 µg ml^−1^. Bacteria were grown in Luria–Bertani (LB) broth or M63 minimal medium supplemented with 1 mM MgSO_4_, 0.2% glucose and 0.5% casamino acids. Where appropriate, IPTG was added to a final concentration of 1 mM for gene expression in *P. aeruginosa*.

### Strain and plasmid construction

Transcriptional fusions were generated by PCR amplification of the putative *cupB* and *cupC* promoter regions, each containing a DNA segment encompassing 472 bp upstream and 137 bp downstream from the translational initiation codon of the *cupB1* gene, and 445 bp upstream and 185 bp downstream from the translational initiation codon of the *cupC1* gene (primers are listed in Table S2). The promoter fragments were cloned into pCR2.1 and after excision by digestion with BamHI and XhoI, subcloned into mini-CTX-*lacZ* vector followed by integration of the appropriate fragment into the PAK chromosome at the *attB* site using established protocols (Hoang *et al*., 2000).

To construct the *rocA2* deletion mutant, PCR was used to generate a 560 bp DNA fragment upstream (Up) from the *rocA2* gene bearing a 3′ BamHI restriction site and a 505 bp DNA fragment downstream (Dw) from the *rocA2* gene bearing a 5′ BamHI site using the UA3045-LA3045 and UB3045-LB3045 oligonucleotide pairs respectively (Table S2). The Up and Dw fragments were used in a three partner ligation into pCR2.1. The resulting 1065 bp Up-Dw DNA fragment was digested from pCR2.1 using SpeI and ApaI and subcloned into the suicide vector pKNG101 (Kaniga *et al*., 1991) digested with SpeI and ApaI. The resulting plasmid pKNGΔ*rocA2* was mobilized into *P. aeruginosa* and the deletion mutants were selected on LB plates containing 5% sucrose and appropriate antibiotics as previously described (Kaniga *et al*., 1991). The double *rocA1-rocA2* deletion mutant was constructed by conjugating pKNGΔ*rocA2* into a *P. aeruginosa rocA1* deletion (Kulasekara *et al*., 2005) and proceeding further as described above. The *rocS2* and *rocA2* genes were obtained from the Gateway library of PAO1 *orfs* (Labaer *et al*., 2004) and cloned into the IPTG-inducible expression vector pMMB67HE42, yielding pMMB67-*rocS2* and pMMB67-*rocA2*.

### Measurements of β-galactosidase activity

M63 medium supplemented with appropriate antibiotics and IPTG (1 mM) was inoculated to OD_600_ 0.1 with overnight cultures of strains carrying *lacZ* transcriptional fusions. Cultures were incubated at 37°C with shaking, and samples were harvested at 6 h post inoculation. β-galactosidase assays were carried out as previously described, and activity was expressed in Miller Units (Miller, 1992).

### Bacterial two-hybrid assay

DNA fragments encoding the protein domains of interest were cloned into plasmids pKT25 and pUT18c, which each encode for complementary fragments of the adenylate cyclase enzyme, as previously described by Karimova and collaborators (Karimova *et al*., 1998). DNA fragments encoding the Hpt domain of RocS2 (RocS2-Hpt), the cytoplasmic domain of GacS (GacS-H1-D1-Hpt) and the D2 domain of RocA2 (RocA2-D2) or GacA (GacA-D2) were amplified by PCR using *P. aeruginosa* PAK genomic DNA. PCR products were cloned into pKT25 (Hpt) and pUT18c (D2) respectively. pKT25 and pUT18c recombinant plasmids were transformed simultaneously into the *E. coli* DHM1 strain, which lacks adenylate cyclase, and screened for positive interactions. Transformants were spotted on MacConkey agar plates (Difco) supplemented with 1% maltose, in presence of 100 µg ml^−1^ ampicillin, 50 µg ml^−1^ kanamycin and 1 mM IPTG. Positive interactions were identified as dark red colonies on MacConkey after 48 h incubation at 30°C followed by 96 h at room temperature. We also used the previously engineered pUT18c derivative encoding RocR-D2 (Kulasekara *et al*., 2005). The positive controls used in the study were pUT18c or pKT25 derivatives encoding the Hpt domain of TorS (TorS-Hpt), the D2 domain of TorR (TorR-D2), the Hpt domain of RocS1 (RocS1-Hpt) and the D2 domain of RocA1 (RocA1-D2) as previously described by Kulasekara and collaborators (Kulasekara *et al*., 2005). Finally, a pKT25 derivative encoding TrpO-D2 was engineered and used as a negative control. For quantitative assays, cells were scraped from the plates and resuspended thoroughly in water. β-galactosidase assays were then carried out in the same manner as for liquid cultures.

### Microarray analysis

For each strain, microarray experiments were performed in triplicate. Independent overnight cultures of *P. aeruginosa* PAK and PAKΔ*rocA2*, both harbouring pMMB67-*rocS2* were resuspended in M63 medium as described above, to OD_600_ 0.1 in the presence of appropriate antibiotics and 1 mM IPTG. Cells were then grown at 37°C with shaking and samples were harvested after 5 h for RNA extraction. Under these conditions, the final OD_600_ of both strains was nearly identical. RNA*later*® (Ambion) was added immediately to the harvested cells to stabilize RNA and prevent degradation. Cell suspensions were then centrifuged at 4°C and RNA extraction was carried out using Promega SV Total RNA Isolation Kit according to manufacturer's protocol. The protocol was modified such that the DNase I digestion step was carried out twice to reduce amount of contaminating DNA. The integrity of RNA preparations was checked using the Agilent 2100 Bioanalyzer and the expression profiling experiment was carried out at the Microarray Facility, Institute of Infection, Immunity and Inflammation, University of Nottingham, as previously published (Rampioni *et al*., 2010). The microarrays were designed to contain oligonucleotide probes for all the PAO1 genes including the small RNA genes and were purchased from Oxford Gene Technology (Oxford, UK). Briefly, for each array, 10 µg of RNA was reverse transcribed and labelled with Cy5-dCTP and 2 µg of genomic DNA was labelled with Cy3-dCTP. Samples were hybridized onto the arrays for 16 h. Scanning of the arrays was performed using the Axon 4000B GenePix Scanner (Molecular Devices, Sunnyvale, USA) and data analysis performed using GeneSpring GX10 (Agilent Technologies, Santa Clara, USA). The array data underwent Lowess normalization and genes of altered expression were determined by passing through cut-offs of both a fold change of 1.5 and a paired *t*-test of *P* = 0.05.

### Quantitative reverse transcription PCR

The same growth conditions as in the microarray analysis described above were used. Quantitative RT-PCR was performed as previously described (Burr *et al*., 2006). Briefly, for first-stranded cDNA synthesis, 200 ng of total RNA was used in a reverse transcription reaction using Invitrogen SuperScript® II Reverse Transcriptase and random hexamers (Applied Biosystems) and reactions were performed according to the manufacturer's protocol. The expression levels of the *mexA* and *mexR* genes were assessed using SYBR Green PCR Master Mix and the 7300 Real Time PCR System apparatus (Applied Biosystems). The primers used for the amplification of *mexA* are mexAup and mexAdown; those for *mexR* are mexRup and mexRdown (Table S2). The gene transcription levels were normalized in each strain to the 16S ribosomal RNA gene (16SrRNA) and expressed as ratios to the values of the PAK strain (set to 1). Samples were assayed in triplicate for each condition.

### Antibiotic susceptibility assays

For antibiotic disc diffusion assays, 1.5% M63 agar (same composition as liquid M63 medium) supplemented with appropriate antibiotics for selection was overlaid with 0.7% M63 soft agar seeded with bacterial strains (PAK or PAKΔ*rocA2* carrying pMMB67-*rocS2* or pMMB67-*rocS1*) as indicated in the individual experiments. Filter discs containing antibiotics as indicated were then placed onto the agar overlay, and plates were incubated at 37°C overnight. Antimicrobial sensitivity was determined by measuring the zone of inhibition around the discs. MICs were determined by serial twofold broth dilution method using M63 minimal media with an inoculum size of 10^5^ cells. Growth was assessed visually after 27 h of static incubation at 37°C. The MIC was defined as the lowest concentration of antimicrobial agent that inhibited visible growth. Cinoxacin and sulfamethoxazole (sulphonamide antibiotic) were from Sigma-Aldrich.
