# Supplementary material for: Two-component regulatory systems in Pseudomonas aeruginosa: an intricate network mediating fimbrial and efflux pump gene expression
Source: Mol Microbiol. 2011 Jan 24;79(5):1353–66. doi: 10.1111/j.1365-2958.2010.07527.x (PMC3083521; doi:10.1111/j.1365-2958.2010.07527.x)
Supplement: Supplementary file 1 [file mmi0079-1353-SD1.pdf]

SUPPORTING INFORMATION

Table S1. The RocA2 regulon: microarray data.

PA number	Gene / Alternate gene name	Predicted function	Fold-change (Overexpression *rocS2*: WT/Δ*rocA2*)
**PA3045**	rocA2	two-component response regulator	17.93
**PA2275**	yahK ;	probable alcohol dehydrogenase (Zn-dependent)	2.869
**PA0248**	pobR ;	probable transcriptional regulator	2.552
**PA3788**		hypothetical protein	2.243
**PA3411**		hypothetical protein	2.116
**PA4182**		hypothetical protein	2.111
**PA1899**	phzA2	probable phenazine biosynthesis protein	2.096
**PA0707**	toxR	transcriptional regulator ToxR	2.046
**PA3593**		probable acyl-CoA dehydrogenase	1.961
**PA4224**	pchG	pyochelin biosynthetic protein PchG	1.855
**PA0510**	nirE ;	probable uroporphyrin-III c-methyltransferase	1.835
**PA2413**	pvdH	L-2,4-diaminobutyrate:2-ketoglutarate 4-aminotransferase, PvdH	1.82
**PA1426**		hypothetical protein	1.736
**PA1569**		probable major facilitator superfamily (MFS) transporter	1.723
**PA0698**		hypothetical protein	1.714
**PA1404**		hypothetical protein	1.71
**PA2358**		hypothetical protein	1.702
**PA1334**		probable oxidoreductase	1.686
**PA5541**	pyrQ	dihydroorotase	1.682
**PA0642**		hypothetical protein	1.667
**PA2418**		hypothetical protein	1.662
**PA2440**		hypothetical protein	1.637
**PA4938**	purA	adenylosuccinate synthetase	1.621
**PA2680**		probable quinone oxidoreductase	1.597
**PA0195**	pntAA	putative NAD(P) transhydrogenase, subunit alpha part 1	1.586
**PA0268**		probable transcriptional regulator	1.585
**PA2683**	tdcB ;	probable serine/threonine dehydratase, degradative	1.585
**PA5013**	ilvE	branched-chain amino acid transferase	1.573
**PA4159**	fepB	ferrienterobactin-binding periplasmic protein precursor FepB	1.571
**PA4121**		conserved hypothetical protein	1.571
**PA0339**		hypothetical protein	1.566
**PA2289**		conserved hypothetical protein	1.558
**PA5297**	poxB	pyruvate dehydrogenase (cytochrome)	1.554
**PA1854**		conserved hypothetical protein	1.549
**PA4183**		hypothetical protein	1.544
**PA4651**		probable pili assembly chaperone	1.54
**PA2665**	ygaA ;	probable transcriptional regulator	1.539
**PA2431**		hypothetical protein	1.537
**PA3723**	yqjM ;	probable FMN oxidoreductase	1.528

**PA3307**		hypothetical protein	1.515
**PA5390**		probable peptidic bond hydrolase	1.514
**PA0723**	coaB	coat protein B of bacteriophage Pf1)	1.514
**PA0636**		hypothetical protein	1.511
**PA5342**		probable transcriptional regulator	1.51
**PA0203**		probable binding protein component of ABC transporter	−389.5
**PA0424**	mexR	multidrug resistance operon repressor MexR	−23.76
**PA0426**	mexB	Resistance-Nodulation-Cell Division (RND) multidrug efflux transporter MexB	−8.42
**PA0427**	oprM	Major intrinsic multiple antibiotic resistance efflux outer membrane protein OprM precursor	−8.229
**PA0425**	mexA	Resistance-Nodulation-Cell Division (RND) multidrug efflux membrane fusion protein MexA precursor	−8.05
**PA1087**	flgL	flagellar hook-associated protein type 3 FlgL	−6.988
**PA0207**		probable transcriptional regulator	−4.138
**PA0202**		probable amidase	−3.862
**PA1892**		hypothetical protein	−2.829
**PA2412**		conserved hypothetical protein	−2.359
**PA2310**		hypothetical protein	−2.338
**PA4507**		hypothetical protein	−2.013
**PA4506**	dppF ;	probable ATP-binding component of ABC dipeptide transporter	−1.962
**PA2135**		probable transporter	−1.924
**PA2670**		hypothetical protein	−1.889
**PA5053**	hslV	heat shock protein HslV	−1.828
**PA5256**	dsbH	disulfide bond formation protein	−1.813
**PA2130**	cupA3	usher CupA3	−1.806
**PA1664**		hypothetical protein	−1.798
**PA1699**	pcr1 ;	conserved hypothetical protein in type III secretion	−1.778
**PA0717**		hypothetical protein of bacteriophage Pf1	−1.765
**PA5191**		hypothetical protein	−1.759
**PA0719**		hypothetical protein of bacteriophage Pf1	−1.727
**PA3406**	hasD	transport protein HasD	−1.699
**PA1893**		hypothetical protein	−1.686
**PA3842**	orf1 ;	probable chaperone	−1.659
**PA0044**	exoT	exoenzyme T	−1.651
**PA0213**	mdcG ;	hypothetical protein	−1.624
**PA0996**	pqsA	probable coenzyme A ligase	−1.623
**PA1708**	popB	translocator protein PopB	−1.61
**PA2891**	atuF	geranyl-CoA carboxylase, alpha-subunit (biotin-containing)	−1.604
**PA1706**	pcrV	type III secretion protein PcrV	−1.599
**PA3126**	ibpA	heat-shock protein IbpA	−1.594
**PA5450**	wzt	ABC subunit of A-band LPS efflux transporter	−1.569
**PA4120**		probable transcriptional regulator	−1.565
**PA2595**		conserved hypothetical protein	−1.559
**PA2303**		hypothetical protein	−1.557
**PA4188**		conserved hypothetical protein	−1.556
**PA4500**		probable binding protein component of ABC transporter	−1.54
**PA1991**		probable iron-containing alcohol dehydrogenase	−1.516

**PA0186**	atsR ;	probable binding protein component of ABC transporter	−1.511
**PA2653**	yuiF ;	probable transporter	−1.507

Table S2. Oligonucleotides used in this study

Oligonucleotide name	Oligonucleotide sequence*
rocA2 deletion	
UA3045	AACGAGCTCCGTGGCTCGGTCAATGAAGA
LA3045	CGCGGATCCGCTCATTGTCTGATTTCCTT
UB3045	CGCGGATCCATCTAAGGGCCGGAACCGAA
LB3045	GCTCTAGACCAGTCGGAATCGGTCAACA
cupC1 promoter	
PromC1up	AGATCGGACCAGATAGAGGT
PromC1down	CCGAGGCTTACGTCGATGGTC
cupB1 promoter	
PromB1up	AGGGTTCTCCTCGTCGTGGG
PromB1down	TTGATATCGCACGCGACTTCG
Bacterial two-hybrid	
rocS2H2up	GCTCTAGAGGCGACCTCTTTCGGACGCGA
rocS2H2down	GGGGTACCGGAAGGGCTCATACCGCAGC
rocA2D2up	GCTCTAGAAAATCAGACAATGAGCCGTA
rocA2D2down	GGGGTACCTCAAACCGAGCTGGGAAAGTAACTG
gacSHDHup	GCTCTAGACAGGATCAAGTCCGAGTTCC
gacSHDHdown	GGGGTACCTGCGCATGGTCAGAGTTCGC
gacAD2up	AAAACTGCAGCGTGATTAAGGTGCTGGTGGTCGACG
gacAD2down	CGGAATTCTATTAGGCGAAGACCTGGCGAATCGCC
trpOD2up	GCCTCTAGAGCGCATGAGTAAGGTGCTGATCGTCG
trpOD2down	GGGGTACCTCAATGGTTGATGCTGCGCAACGCCCCGGT
qPCR	
mexAup	TGAACGGCATCATCCTCAAG
mexAdown	TGGTAGTCGGCCTCGTAGGT
mexRup	CGCCCGACGTCCATGTAT
mexRdown	CGGGTGATCAGTGCCTTGT
16SrRNAup	CGCCGTAAACGATGTCGACTA
16SrRNA down	TTAACCTTGCGGCCGTACTC

*Oligonucleotides are presented in the orientation 5’-3’.

Fig. S1. Domain organization of paralogous Roc proteins of *P. aeruginosa*. This representation is based on predictions of Protein families' database of alignments (Pfam). Pfam accession for each domain is as follows: Sbp3 (PF00497), PAS (PF00989, PF08448), HisKA (PF00512), HATPase_c (PF02518), RR (PF00072), Hpt (PF01627), GerE (PF00196) and EAL (PF00563). The sensor kinases RocS1 and RocS2 domains named H1, D1 and H2 refer to the HisKA/HATPase_c, RR and Hpt domains, respectively. For the response regulators, the RR and GerE are referred to as the D2 and helix-turn-helix (HTH) domains, respectively (Kulasekara *et al.*, 2005). TM indicates the location of the transmembrane segment of the sensor kinases. RocS1 and RocS2 are paralogous proteins with 45% identity, 63% similarity and RocA1 and RocA2 are also paralogous proteins with 59% identity, 73% similarity. The RocR response regulator is also shown with a D2 domain and an EAL-containing domain with phosphodiesterase activity.

Fig. S2. Activity of the *cupB* promoter in different *P. aeruginosa* strains. The strains tested are the wild type PAK, the *rocA2* mutant (PAKΔ*rocA2*), the *rocA1* mutant (PAKΔ*rocA1)*, and the *rocA1*/*rocA2* mutant, all carrying the transcriptional *cupB-lacZ* fusion on the chromosome at the *attB* site. Where indicated the strain contains a plasmid expressing the *rocS1* gene (pMMB67-*rocS1*), or empty vector (pMMB67EH). All data presented are the average of biological duplicates and error bars indicate one standard deviation of the mean. β-galactosidase activity is expressed in Miller units.

Figure S1

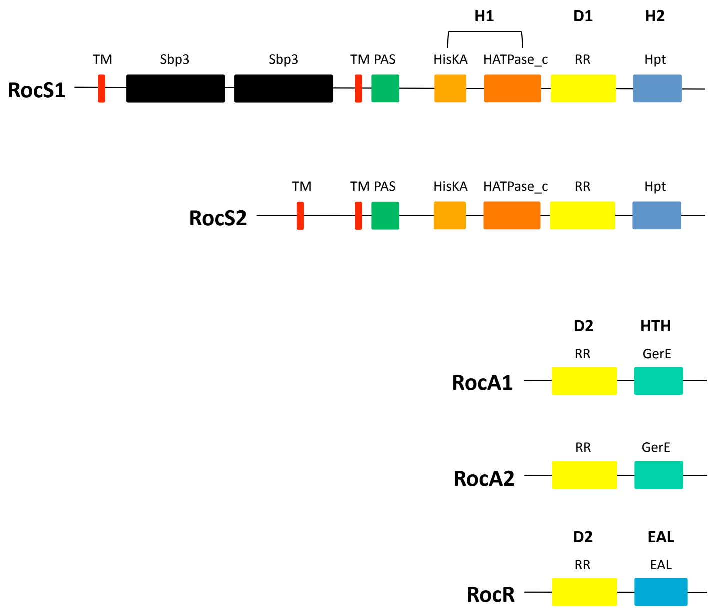
H1
D1
H2
TM
Sbp3
Sbp3
TM PAS
HisKA
HATPase_c
RR
Hpt
RocS1
TM
TM PAS
HisKA
HATPase_c
RR
Hpt
RocS2
D2
HTH
RR
GerE
RocA1
RR
GerE
RocA2
D2
EAL
RR
EAL
RocR

Figure S2

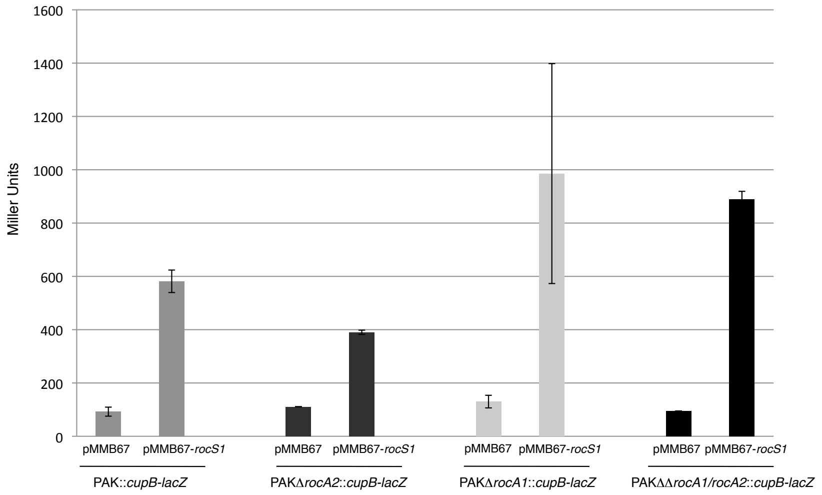

1600
1400
1200
1000
800
600
400
200
0
Miller Units
pMMB67
pMMB67-rocS1
PAK::cupB-lacZ
pMMB67
pMMB67-rocS1
PAKΔrocA2::cupB-lacZ
pMMB67
pMMB67-rocS1
PAKΔrocA1::cupB-lacZ
pMMB67
pMMB67-rocS1
PAKΔΔrocA1/rocA2::cupB-lacZ
